# Assessing Perceptions and Behaviors Related to Vaping Nicotine: The Nicotine Addiction Perceptions Scale

**DOI:** 10.1177/1179173X251336468

**Published:** 2025-04-26

**Authors:** Allison A. Temourian, Anna V. Song, Anna E. Epperson

**Affiliations:** 1 Center for Tobacco Control Research and Education, 8785 University of California, San Francisco, CA, USA; 2 Nicotine and Cannabis Policy Center, 204804 University of California, Merced, CA, USA; 3 Department of Psychological Sciences, School of Social Sciences, Humanities, & Arts, 204804 University of California, Merced, CA, USA

**Keywords:** perceptions of nicotine addiction, scale development, e-cigarettes

## Abstract

**Background:**

Existent research examining perceptions of nicotine addiction use largely surface level items that fail to address the complexity of nicotine addiction. Additional investigation is needed to better understand people’s perceptions of nicotine addiction and whether these align with measures of nicotine dependence. Understanding these perceptions about nicotine addiction may help provide insight into vaping intentions and behavior. This study examines the validity of the Nicotine Addiction Perceptions (NAP) scale, a novel measure designed to assess perceptions of addictive vaping behavior that aligns with the clinical dimensions of tobacco use disorder.

**Methods:**

Data were collected from U.S. adults via Prolific (n = 549). As part of scale development and validation a confirmatory factor analysis and psychometric evaluation was conducted. The NAP’s reliability, convergent, discriminant, and criterion validity were established.

**Results:**

A five-factor solution returned acceptable fit on all model indices (RMSEA = 0.050; CFI = 0.994; TLI = 0.993). The NAP was significantly related to assessments of perceived risk, 6 month quit intentions, the number of quit intentions over the past year, and past 30-day e-cigarette use (*P’s* < .05). Findings also indicate support for discriminant validity.

**Conclusions:**

Findings suggest that for most, perceptions of nicotine addiction may not fully align with the clinical criteria of addiction, which may be due to the lack of education surrounding the clinical definition. Future research examining perceptions of nicotine addiction can utilize the NAP scale to better understand people’s understanding of addiction and its relationship to vaping-related behavior.

## Introduction

Prevalence of tobacco use remains problematic^1^ and is one of the most common addictions.^2-4^ In a nationally representative sample of United States (U.S.) adults who smoke (n = 6793), 85% of daily smokers and 44% of non-daily smokers met the diagnostic criteria of addiction.^5^ Faced with declining cigarette consumption as a result of tobacco control measures, tobacco companies are increasingly promoting alternative methods of nicotine consumption, such as electronic cigarettes (e-cigarettes).^6,7^ These products are presented as “risk reductive” primarily because they are reported to allow a consumer to avoid negative consequences of combustion which includes inhalation of particles and hydrocarbons that can cause severe tissue damage.^8^ However, these alternative tobacco products – which still contain nicotine – have been shown to hold their own health risks including increased risk of damaged lung function, heart disease, and cancer^9^ related to the ingestion of heavy metals and aerosols, volatile organic compounds, and toxic flavorings,^10,11^ and do not reduce the harm of nicotine addiction itself.^9,11^

In a nationally representative sample of approximately 30 000 U.S. adults, 6.5% reported current use of e-cigarettes in 2023.^12^ Initially touted as a cessation tool for combustible cigarette users,^13^ e-cigarette use has permeated into other groups, including people who have never used combustible cigarettes.^14-16^ Given the changing landscape of tobacco products over the past decade, it is probable that cigarette use may no longer be the only major prototype of nicotine consumption, and that vaping may complement cigarette use.^13^

### Current Assessments of Perceptions of Nicotine Addiction

Single item assessments of perceptions of nicotine addiction are highly varied between researchers.^17^ Much of this work typically uses one comparative item that assesses perceived addictiveness of e-cigarettes relative to combustible cigarettes, despite the addictive component of all tobacco products being nicotine.^18,19^ Thus, it is difficult to meaningfully understand how people perceive nicotine addiction to e-cigarettes as this measure is dependent upon the measure of perceived cigarette addiction. Even in cases where item phrasing may be identical, response options given to participants vary across studies and the lack of standardization of this construct hinders replicability and contributes to the cursory understanding researchers have developed when assessing perceptions of nicotine addiction.^17^ Moreover, assessments of perceptions of nicotine addiction must be distinguished from diagnostic measures of nicotine addiction as the former assesses people’s beliefs surrounding addiction and the latter assesses addictive behavior.

Nicotine addiction, or dependence, has physical, psychological, and social elements, all of which can influence use and cessation.^20^ Physical aspects of nicotine addiction can include impaired cognitive performance when deprived of nicotine or weight loss due to decreased appetite.^21^ Research has also found that nicotine can cause permanent changes in the brain that can make the individual more dependent.^22^ Nicotine provides reduction in anxiety and stress when in withdrawal, which can occur throughout the day for the smoker if they are not smoking frequently, and includes psychological symptoms such as anxiety or depressed mood.^20^ Finally, social aspects of nicotine dependence can include increased positive social functioning while smoking, thus perpetuating dependence,^23^ but poorer social interactions when abstaining.^24^ For example, an established tobacco user may be asked to avoid smoking around others (eg, friends, loved ones, etc.) and may isolate themselves in order to continue smoking, reducing their social circle and negatively impacting their well-being. Using tobacco against one’s will is a clinically recognized aspect of addiction, yet despite this understanding, psychometric evaluation of people’s appraisal of nicotine addiction is lacking and scales that do exist fail to address the complexity of nicotine addiction.^17^ To assess one’s susceptibility of becoming addicted to nicotine as well as their severity (ie, the strength of addiction were nicotine used), researchers must first understand how the lay person (ie, general public) appraises singular behaviors. For example, does the lay person think that the only sign of nicotine addiction is craving tobacco? Or is a sign of nicotine addiction a confluence of multiple behaviors, and if so, what are those behaviors? Concerns related to nicotine addiction among those with little to no experience with vaping may elucidate their reasoning behind initiating e-cigarettes. Similarly, understanding how an established e-cigarette user perceives addiction may help in tailoring cessation interventions (eg, highlighting dimensions of addiction that may not be as commonplace). This study aims to develop a scale that not only encapsulates the perceived breadth of experiences when addicted to nicotine, but also assesses the intensity of those experiences. By measuring perceptions of nicotine addiction in line with clinical dimensions, researchers can determine how perceptions of these dimensions of addiction contribute to health decision making – both *which* dimensions contribute and *how much* they contribute.

### Diagnostic Assessments of Nicotine Addiction

Assessments of nicotine addiction (ie, dependence) between clinicians and researchers are highly varied. According to the Diagnostic and Statistical Manual 5^th^ edition (DSM-5),^25^ manifestation of tobacco use disorder (ie, addiction) involves experiencing at least two of 11 dimensions within a 12-month period, with more dimensions experienced equating to a more severe addiction. These 11 dimensions include: (1) consuming substance in larger amounts or over a longer period than intended, (2) a persistent desire or unsuccessful efforts to reduce or control substance use, (3) a great deal of time is spent in activities necessary to obtain or use substance, (4) craving of substance, (5) recurrent substance use resulting in failure to fulfill major role obligations, (6) continued substance use despite persistent or recurrent social or interpersonal problems caused or exacerbated by the effects of the product, (7) important social, occupational, or recreational activities are given up or reduced because of substance use, (8) recurrent substance use in physically hazardous conditions, (9) continued substance use despite knowledge and experience of psychological or physical harm associated with substance use, (10) tolerance of substance, and (11) withdrawal from substance. These dimensions can be largely categorized into either (1) behavioral changes, or (2) affective symptoms.

In contrast, researchers typically utilize scales such as the Nicotine Dependence Syndrome Scale (NDSS)^26^ or the Fagerström Test for Nicotine Dependence (FTND).^27^ The FTND and NDSS are ubiquitous in psychological research, yet items found in these scales are not aligned with clinical guidelines, let alone each other. The FTND determines addiction severity based on items such as the amount of tobacco consumed per day and how quickly after waking one smokes. Conversely, the NDSS assesses addiction severity based on a host of items, including questions related to tolerance and reduced social activity in order to smoke. Though both scales have been validated to assess addiction severity in research settings, neither scale comprehensively aligns with clinical guidelines (ie, DSM-5). Moreover, there is no overlap in items between the NDSS and FTND, making it difficult to compare scores. The incongruencies in measurement of addiction severity and lack of emphasis on how people *conceptualize* nicotine addiction has led to a cursory understanding of what addiction means for the lay person.

### Current Study Aims

To better understand addiction perceptions, the disentanglement of the physical, psychological, and social components of nicotine addiction are warranted. Specifically, when considering nicotine addiction, do people primarily focus on withdrawal symptoms, craving, or inability to quit smoking despite the social and economic costs? Understanding how the lay person views and thinks about nicotine addiction is instrumental in developing and refining current targeted interventions for both prevention and intervention-based research. Using a previously established scale development framework^28^ and based on the clinical dimensions of tobacco use disorder, this study aimed to psychometrically evaluate and validate a novel scale.

## Methods

### Participants

In December 2023, participants were recruited using Prolific, an online survey platform comprised of active participants that have been screened and verified prior to participation.^29^ The survey was hosted on Qualtrics^30^ an online survey building platform. To be eligible for inclusion, participants were required to be ≥ 18 years old and English speakers living in the U.S. Both e-cigarette users and non-users were recruited to be in the study. Perceptions of nicotine addiction among both current and non-e-cigarette users can provide insight into their decision-making processes by highlighting the aspects of addiction most salient for them. For their participation, participants received $4.00 credit to their Prolific account. This study was deemed exempt by the University of California, Merced Institutional Review Board (UCM2023-28) as the research only involved interactions via survey procedures, and the information obtained is recorded by investigators in such a manner that the identity of human subjects cannot be readily ascertained, directly or through identifiers linked to the subjects. Prior to the survey, participants received a written consent form that described the study, and denoted their consent to participate in the current study before moving onto the survey. This study was not preregistered.

To determine the minimum number of participants needed to test study hypotheses, an a-priori power analysis was run using G*power.^31^ Informed by quantitative literature, we set power at .95 to reduce the likelihood of detecting erronenous significance based on small sample size,^32^ and had a small effect size of 0.11 given that our power is relatively restrictive,^33^ to which G*power recommends a total sample size of 253. Thus, the obtained sample of n_e-cigarette users_ = 277 & n_non-e-cigarette users_ = 275, was adequate to test study hypotheses. After removing missing data (n = 3), the final sample for n_e-cigarette users_ = 274.

### Measures

#### Nicotine Addiction Perceptions (NAP) Scale

Participants responded to 36 items assessing their perceptions of nicotine addiction (see Supplemental Material 1) which was part of a larger survey assessing risk behavior (see Data Availability). In collaboration with an expert in tobacco control,^34-36^ a new scale was developed, with each item created using the 11 DSM-5 dimensions of tobacco use disorder as a guiding framework. Before settling on a finalized version, the NAP scale directions and items underwent the cognitive interview process with a separate, independent sample to determine item comprehension and improve its readability. The cognitive interview sample were comprised of active e-cigarette users and non-users. In its finalized version, the prompt read, “Below we are going to give you a list of behaviors related to nicotine based vapes/e-cigarettes. On a scale from 1-5, how important are any of these behaviors in telling you a person is addicted to vapes/e-cigarettes?”. Participants indicated their perceptions of addiction to nicotine with items such as “Having difficulty with quitting vaping” and “Giving up hobbies in order to vape” on a five-point scale from 1 (not at all important) to 5 (extremely important).

#### Perceptions of Harm and Addiction

To measure perceptions of e-cigarette harm to one’s own health, participants were asked, on a five-point Likert type scale with 1 = very unlikely and 5 = very likely, “If you continue to smoke e-cigarettes, how likely is it that you will harm your own health?”; non-users were asked, “If you were to smoke e-cigarettes, how likely is it that you will harm your own health?”. To measure perceptions of e-cigarette harm to other’s health, users were asked, on a five-point Likert type scale with 1 = very unlikely and 5 = very likely, “If you continue to smoke e-cigarettes, how likely is it that you will harm someone else’s health with second hand vape smoke?”; non-users were asked, “If you were to smoke e-cigarettes, how likely is it that you will harm someone else’s health with second hand vape smoke?”.

Adapted from previously validated items in the Population Assessment of Tobacco and Health (PATH) study that have been shown to relate to smoking-related behavior,^37^ participants were asked about comparative e-cigarette harm with the question, “Compared with cigarettes, how harmful are e-cigarettes to a person’s health”? Response options followed a Likert scale format from 1 = much less harmful than cigarettes to 5 = much more harmful than cigarettes. Perceptions of perceived e-cigarette addictiveness was assessed with the question “If you continue to smoke e-cigarettes, how likely is it that you will become addicted?”, response options were on five-point Likert type scale with 1 = very unlikely and 5 = very likely. Non-users were asked, “If you were to smoke e-cigarettes, how likely is it that you will become addicted?”.

#### Tobacco Use and Cessation

The full sample consisted of adults who did not use an e-cigarette (non- e-cigarette user) and adults who actively used an e-cigarette (current e-cigarette user). Some non- e-cigarette users had tried an e-cigarette (but no more than 20 times in their lifetime; defined by Prolific and verified during the survey process). Participants classified as current e-cigarette users (defined as using an e-cigarette in the past 30 days), were also asked questions about their use and cessation-related behavior. Current e-cigarette users were asked to report the number of days they vaped in the past 30 days from 0 days to 30 days. This was separated into three categories (1 = 1-10 days, 2 = 11-24 days, 3 = 25 or more days). Participants were asked about nicotine dependence using the NDSS, a 19-item questionnaire previously validated,^26^ where they indicated how well the following statements described them from 1 = not at all true to 5 = extremely true. Language for the scale was adapted for e-cigarette use (eg, “Whenever I go without vaping for a few hours, I experience craving”). Participants also answered questions from the FTND, a six-item instrument that has been previously validated and assesses the physical intensity of nicotine addiction using a summated score where 0-2 = low dependence, 3-4 = low to moderate dependence, 5-7 = moderate dependence, 8+ = high dependence.^27^ Language for the scale was also adapted for e-cigarette use (eg, “Which vaping session would you hate most to give up”?). Current e-cigarette users were asked to report how soon they use their e-cigarette/vape after waking (1 = within 5 minutes, 2 = 5-30 minutes, 3 = 31-60 minutes, 4 = longer than 60 minutes) and how many times a day they used an e-cigarette device (0 = 0 times a day – 30 = 30 or more times a day). This was separated into three categories (1 = 1-9 times a day, 2 = 10-24 times a day, 3 = 25 or more times a day). The FTND was scored in line with established scoring guidelines.

E-cigarette users were asked their primary reason or motivation for using an e-cigarette (including non-e-cigarette users who had some experience using an e-cigarette).^37^ Participants could only select one option which included: to quit smoking, to reduce smoking, to use when I cannot or am not allowed to smoke, enjoyment, or curiosity. Participants were asked whether their e-cigarette liquids contained nicotine (0 = no, 1 = yes; including non-e-cigarette users who had some experience using an e-cigarette). Finally, users were asked about their intentions to quit in the next 30 days and 6 months (0 = no, 1 = yes) and number of quit attempts in the past 12 months for at least 24 h (0 = no, 1 = yes). Additionally, participants were asked the number of times they’ve attempted to quit vaping in the past 12 months (0-12 or more).

#### Covariates

##### Mental Health

Participants completed the PROMIS short-form anxiety and depression questionnaires, which have both been previously validated.^38^ The PROMIS questionnaires utilize a five-point Likert scale ranging from 1 (never) to 5 (always). Depression and anxiety scores were highly correlated (Pearson’s *r* = 0.80), so we created a composite score by averaging depression and anxiety scores together. Higher scores indicated experiencing greater mental distress over the previous seven days.

##### Demographics

Participants were asked demographic questions (gender, race/ethnicity, income, education, and age). For *gender,* participants were considered male if they selected male or female-to-male transgender, and female if they selected female or male-to-female transgender. Other gender identities (non-binary or declined to answer) were not included in analyses due to low cell count (n = 14). Gender was dichotomized (males = 0, females = 1). For *race/ethnicity*, participants were either coded as non-Hispanic/Latinx (NH) White, NH African-American/Black, Hispanic/Latinx (Hispanic), or NH Other. For *education*, respondents were either coded as having a high school degree or less, some college education, a Bachelor’s degree, or graduate degree. *Income* was categorized into five groups (<$25,000; $25,001-$50,000; $50,001-$75,000; $75,000- $100,000; $100,001+). Demographic characteristics of the sample can be found in Supplemental Material 2.

### Analytic Plan

#### Confirmatory Factor Analysis

A confirmatory factor analysis (CFA) of the six-factor structure based on the team’s initial work developing this scale was conducted (see Supplemental Material 3). Respondents with missing values were omitted from analysis (n = 3), after ensuring data were missing completely at random (*P* = 0.1099).^39^ Final factor structure was dependent upon how well items loaded onto their respective factor and whether enough items loaded onto a single latent factor.^40^ After assessing multivariate normality using Mardia’s test of normality, we utilized a mean- and variance-adjusted weighted least squares (WLSMV) estimation method which is preferred as its properties are less biased, there is small sampling variation in estimated factor loadings compared to other estimation methods,^41,42^ and modification indices and associated values can be interpreted in the same manner as CFA with continuous data.^43^ To allow factor loadings to be estimated freely, we fixed latent factor variance to one.

#### Psychometric Assessment- Item Response Theory (IRT)

With rating scale data, item difficulty can be interpreted as the ease in which respondents can endorse a particular item, rather than the percentage of people who answer an item correctly.^44^ Item discrimination refers to an item’s ability to distinguish between individuals with lower and higher scores on the latent construct, with higher scores indicating greater relevance of the item being measured by the latent trait.^45,46^ Item removal was primarily dependent upon difficulty and discrimination scores. Given this is the first attempt at creating a scale to assess perceptions of nicotine addiction, we employed a more conservative approach to retaining items that were highly correlated (see Supplemental Material 4). We assessed internal consistency of the NAP (ie, reliability) using McDonald’s omega (ω) and interpreted findings according to conventional guidelines: 0.9+ = excellent reliability, 0.8-0.9 = good reliability, 0.7-0.8 = acceptable reliability.^47,48^

#### Convergent, Discriminant, and Criterion Validity

We conducted Pearson’s correlations to assess convergent validity between perceived harm to health, harm to other’s health, likelihood of becoming addicted, comparative harm, and the NAP scale among all study participants. Additionally, we conducted Pearson’s correlations to assess discriminant validity between the NDSS, FTND, and NAP among active e-cigarette users. We conducted various regressions to assess criterion validity among active e-cigarette users. Specifically, we conducted binary logistic regressions to analyze 6-month and 30-day quit intentions, and whether a quit attempt had occurred over the past 12 months. Initial analyses for the number of quit attempts over the past 12 months indicated some non-normal data, particularly skewed to the right. To account for the over-dispersion and excess zero responses, we conducted a negative binomial regression in which the Incident Rate Ratio (IRR) refers to the factor change in the outcome variable for each unit increase in the predictor variable.^49^ Finally, we conducted linear regressions to analyze how soon after waking one uses their e-cigarette, daily e-cigarette use, and past 30-day e-cigarette use. For all regression analyses, we controlled for comparative e-cigarette harm, current combustible cigarette user status (yes/no), nicotine dependence scores (NDSS), primary reason for using e-cigarettes, e-cigarette composition, mental health status, and demographic variables. Data were analyzed using Rstudio 2023.06.1.

## Results

In a general U.S. adult sample, the average age of participants was 39.56 years (SD = 12.66 years; Supplemental Material 2), and roughly half were male (51.37%). Most identified as NH White (62.48%). The most commonly reported reason for using an e-cigarette device was for enjoyment (29.4%), followed by curiosity (28.39%), to quit (19.6%) or reduce (14.82%) smoking, and for use when one cannot or is not allowed to smoke (6.03%). A majority of respondents reported using an e-cigarette that contained nicotine (76.84%).

### Confirmatory Factor Analysis

In the current sample, the six-factor solution suggested acceptable model fit on CFA indices: χ2(284, N = 549) = 766.528, *P* < .001; robust Standardized Root Mean Square Residual (SRMR) = 0.044 (scaled SRMR = 0.044; recommended good fit < 0.08), robust Root Mean Square Error of Approximation (RMSEA) = 0.036 (90% CI = 0.033, 0.039; scaled RMSEA = 0.056 (90% CI = 0.051, 0.060; recommended good fit ≤ 0.06), robust Comparative Fit Index (CFI) = 0.991 (scaled CFI = 0.794; recommended value ≥ 0.90), robust Tucker Lewis Index (TLI) = 0.989 (scaled TLI = 0.765; recommended value ≥ 0.90). Standardized item factor loadings can be found in Table 1.

**Table 1.** Confirmatory Factor Analysis Item Loadings.

Factor	Item	Six-factor loading	Five-factor loading
1. Continued use despite negative consequences			
Vaping in places that might cause a fire	21	0.622	0.611
Continued vaping even after experiencing negative health effects from vaping	22	0.734	0.728
Continued vaping even though they are aware it is bad for them	23	0.817	0.829
Continued vaping even though it causes conflict in their familial relationships	24	0.814	0.820
Continued vaping even though it causes conflict in their relationships with friends	25	0.831	0.829
Continued vaping even though it causes conflict in their romantic relationships	26	0.852	0.857
2. Withdrawal			
Craving a vape when they are not vaping	11	0.798	0.803
Needing to vape more to get the same buzz	27	0.780	0.780
Becoming irritable when they do not vape	28	0.864	0.866
Being on edge when they do not vape	29	0.855	0.856
Having a hard time concentrating when they do not vape	30	0.882	0.881
Having trouble sleeping when they do not vape	31	0.849	0.841
3. Tolerance			
Vaping more now than when they first started	1	0.847	0.843
Taking more puffs/hits throughout the day	2	0.883	0.885
Taking more time per day to vape	3	0.917	0.919
4. Social impact			
Vaping gets in the way of their daily life	12	0.888	0.892
Vaping negatively impacts their work productivity	13	0.861	0.860
Person gives up their social life in order to vape	17	0.899	0.899
Giving up part of their job because of vaping	18	0.858	0.860
Giving up hobbies because of vaping	19	0.828	0.824
5. Consistent desire to quit/reduce use			
Often thinking about quitting vaping	6	0.834	0.833
Often thinking about reducing their vaping	7	0.818	0.815
Constantly wanting to quit vaping	8	0.895	0.898
6. Substitution to other behavior			
Eating more when they do not vape	34	0.812	—
Drinking more alcoholic beverages when they do not vape	35	0.859	—
Drinking less alcoholic beverages when they do not vape	36	0.250	—

Note. Factor loadings are standardized.

**Figure 1.**
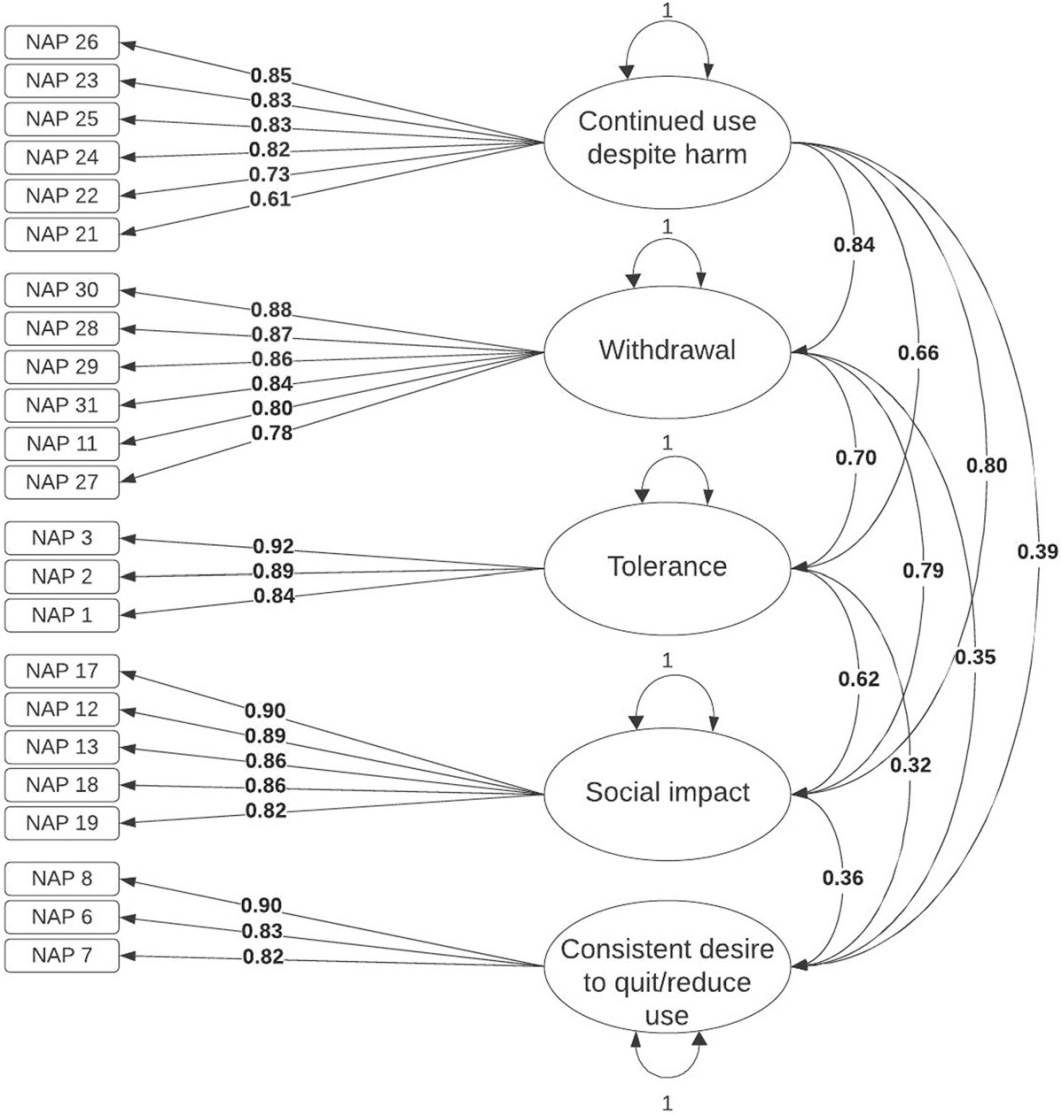
Five Factor Solution Diagram.

After removing a problematic item, “Drinking less alcoholic beverages when they do not vape,” due to its poor factor loading (0.250) and removing Factor 6 entirely due to an insufficient number of items, we re-analyzed the CFA with a five-factor solution. This resulted in better model fit indices: χ2(220, N = 549) = 518.808, *P* < .001, robust SRMR = 0.041 (scaled SRMR = 0.041), robust RMSEA = 0.030 (95% CI = 0.026, 0.033; scaled RMSEA = 0.050, 95% CI = 0.044, 0.055), robust CFI = 0. 994 (scaled CFI = 0.854), robust TLI = 0.993 (scaled TLI = 0.832). Standardized item factor loadings can be found in Table 1, and a diagrammatic representation can be found in Figure 1.

### Psychometric Evaluation of the NAP Scale – IRT Findings

For item difficulty, respondents were likely to endorse item 18 using higher response options (“Giving up part of their job because of vaping; see Table 2), but less likely to endorse item seven (“Often thinking about reducing their vaping). In this regard, participants were likely to agree that item 18 is reflective of someone experiencing addiction. In contrast, participants were not likely to agree that item seven reflects an individual experiencing addiction. Item discrimination values ranged from 0.44 to 0.82 (see Table 2). Items six, seven, and eight had the lowest discrimination scores that ranged from 0.44 to 0.50. These items comprise Factor 5, which was labeled “Consistent Desire to Quit/Reduce Use”. These results suggest that these items were not effective at differentiating between individuals who perceived these vaping behaviors as more indicative of addiction vs less indicative of addiction. The two items with the highest item discrimination were item 30 (0.82) and 17 (0.81), which comprised factors two (labeled “Withdrawal”) and four (labeled “Social Impact”), respectively. These items were effective in differentiating between individuals who perceive these vaping behaviors as more indicative of addiction vs less indicative of addiction. Using McDonald’s omega, the NAP was found to have high internal consistency, with an omega coefficient of 0.958. All subscales had an omega coefficient greater than 0.89.

**Table 2.** Item Analysis.

	Sample.SD	Item.total	Item.Tot.woi	Difficulty (mean)	Discrimination	Item.Reliab	Item.Rel.woi
NAP_1	1.08	0.67	0.63	3.72	0.67	0.72	0.68
NAP_2	1.04	0.70	0.66	3.84	0.70	0.72	0.69
NAP_3	1.06	0.72	0.69	3.81	0.72	0.77	0.74
NAP_6	1.13	0.46	0.40	3.15	0.46	0.52	0.46
NAP_7	1.10	0.44	0.39	3.11	0.44	0.48	0.43
NAP_8	1.20	0.50	0.44	3.33	0.50	0.60	0.53
NAP_11	1.08	0.76	0.73	4.08	0.76	0.82	0.79
NAP_12	1.08	0.80	0.78	4.28	0.80	0.87	0.84
NAP_13	1.07	0.78	0.75	4.23	0.78	0.83	0.80
NAP_17	1.08	0.81	0.78	4.24	0.81	0.87	0.85
NAP_18	1.08	0.78	0.75	4.33	0.78	0.84	0.81
NAP_19	1.12	0.75	0.72	4.14	0.75	0.84	0.81
NAP_21	1.31	0.61	0.56	3.72	0.61	0.80	0.73
NAP_22	1.17	0.70	0.67	3.70	0.70	0.82	0.78
NAP_23	1.04	0.78	0.75	4.16	0.78	0.81	0.79
NAP_24	1.09	0.77	0.74	3.95	0.77	0.84	0.81
NAP_25	1.10	0.78	0.75	3.88	0.78	0.86	0.83
NAP_26	1.07	0.80	0.78	3.96	0.80	0.86	0.83
NAP_27	1.14	0.73	0.70	3.97	0.73	0.84	0.80
NAP_28	1.02	0.80	0.78	4.12	0.80	0.82	0.80
NAP_29	1.05	0.79	0.77	4.07	0.79	0.84	0.81
NAP_30	1.06	0.82	0.80	4.02	0.82	0.87	0.85
NAP_31	1.08	0.79	0.76	4.05	0.79	0.85	0.82

Note. Mean inter-item-correlation = 0.480. NAP = Nicotine Addiction Perceptions.

### Establishing Validity of the NAP Scale

A Pearson’s correlation between the NAP, harm perceptions, and addiction perceptions provided evidence of convergent validity (see Table 3). A Pearson’s correlation between the NAP, FTND, and NDSS provided evidence of discriminant validity (see Table 3). For criterion validity, the association between NAP, quit intentions and past quit attempts, and past month use was examined. For every unit increase in perceptions of nicotine addiction, intentions to quit using e-cigarettes in the next 6 months increased (OR = 1.03, 95% CI: 1.01, 1.04; *P =* .007; Table 4). Participants who perceived vaping behaviors as more addictive were more likely to report future intentions to quit vaping. For each unit of increased perception of nicotine addiction, the number of quit attempts over the past 12 months decreased by a factor of 0.99 (95% CI [0.98, 1.00]). Participants who perceived vaping behaviors as more addictive were likely to have attempted fewer quit attempts over the past 12 months. For every unit increase in perceptions of nicotine addiction, the number of days vaped in the past 30 days increased (b < 0.01, *P* = .039). Participants who perceived vaping behaviors as more addictive were likely to have vaped more over the past 30 days. Full regression results can be viewed in Supplemental Material 5.

**Table 3.** Correlation Table.

Variable	1	2	3	4	5	6	7
1. NAP	—						
2. Harm to health	.2893**	—					
3. Harm to others’ health	.2085**	.5862**	—				
4. Comparative harm	.1479*	.4029**	.3732**	—			
5. Likelihood of becoming addicted	.0994*	.3172**	.1838**	−.0564	—		
6. FTND	.0372	−.0348	−.0312	−.1679*	.3316**	—	
7. NDSS	.0807	−.0220	.1720	−.0914	.1862*	.0019	—

Note. NAP = Nicotine Addiction Perceptions; FTND: Fagerström Test of Nicotine Dependence; NDSS = Nicotine Dependence Syndrome Scale.

*P < .05. **P < .001.

**Table 4.** Regression Results with All Covariates.

Variable	6-month quit intentions (n = 268)	Thirty day quit intentions (n = 268)	Tried quitting in past 12 months (n = 270)	Number of quit attempts over past 12 months (n = 270)	Time to first e-cigarette after waking (n = 270)	Daily e-cigarette use (n = 269)	Monthly e-cigarette use (n = 270)
	Odds ratio (95% CI)			Incident rate ratio (95% CI)	Unstandardized b (95% CI)		
Predictors							
Current cigarette user	0.83 (0.45, 1.55)	0.78 (0.30, 2.04)	0.75 (0.41, 1.37)	1.00 (0.66, 1.52)	0.08 (−0.18, 0.33)	−0.05 (−0.25, 0.15)	0.01 (−0.17, 0.18)
Uses e-liquid that contains nicotine	1.90 (0.84, 4.29)	1.78 (0.51, 6.21)	1.55 (0.73, 3.30)	0.82 (0.48, 1.38)	**−0.49 (-0.81, -0.17)**	0.24 (−0.01, 0.49)	**0.50 (0.28, 0.72)**
Comparative harm	**1.47 (1.05, 2.06)**	**1.89 (1.16, 3.08)**	**1.57 (1.12, 2.20)**	**1.62 (1.28, 2.05)**	0.09 (−0.06, 0.23)	−0.10 (−0.21, 0.01)	**−0.15 (-0.25, -0.05)**
Nicotine addiction perceptions (NAP)	**1.03 (1.01, 1.04)**	1.02 (0.99, 1.04)	0.99 (0.98, 1.01)	**0.99 (0.98, 1.00)**	<0.01 (−0.00, 0.01)	<0.01 (−0.00, 0.01)	**<0.01 (<0.01, 0.01)**
Nicotine dependence (NDSS)	1.00 (0.93, 1.08)	1.05 (0.94, 1.17)	1.02 (0.95, 1.10)	**1.08 (1.03, 1.14)**	−0.03 (−0.06, <0.01)	−0.01 (−0.03, 0.02)	−0.01 (−0.03, 0.01)
Reason for using e-cigarettes (ref: enjoyment)	1.00	1.00	1.00	1.00	1.00	1.00	1.00
To quit smoking	2.10 (0.98, 4.49)	2.23 (0.68, 7.31)	0.90 (0.43, 1.88)	**2.00 (1.17, 3.40)**	**−0.67 (-0.98, -0.35)**	**0.28 (0.03, 0.52)**	**0.25 (0.03, 0.46)**
To cut down on smoking	2.08 (0.97, 4.48)	**3.70 (1.16, 11.75)**	1.63 (0.77, 3.44)	1.15 (0.67, 1.97)	−0.29 (−0.61, 0.03)	−0.03 (−0.27, 0.22)	0.09 (−0.13, 0.31)
To use when cannot or are not allowed to smoke	1.45 (0.49, 4.31)	3.22 (0.68, 15.13)	**3.19 (1.11, 9.18)**	1.75 (0.83, 3.68)	−0.33 (−0.79, 0.13)	0.06 (−0.30, 0.41)	0.08 (−0.23, 0.40)
Curiosity	2.05 (0.48, 8.74)	**25.09 (3.57, 176.30)**	0.51 (0.12, 2.23)	1.04 (0.39, 2.72)	−0.52 (−1.16, 0.12)	0.17 (−0.34, 0.69)	0.11 (−0.33, 0.55)
Some other reason	-	-	2.32 (0.13, 42.58)	<0.01 (0, <0.01)	−0.70 (−2.05, 0.64)	0.76 (−0.27, 1.79)	0.68 (−0.24, 1.60)
Mental health status	1.27 (0.95, 1.70)	1.07 (0.70, 1.63)	1.20 (0.90, 1.59)	**1.24 (1.01, 1.53)**	−0.07 (−0.19, 0.05)	0.04 (−0.06, 0.13)	**−0.09 (-0.18, -0.01)**
Age	1.00 (0.97, 1.03)	0.96 (0.92, 1.00)	0.98 (0.95, 1.00)	**0.98 (0.96, 1.00)**	0.00 (−0.01, 0.01)	−0.00 (−0.01, 0.01)	−0.01 (−0.01, <0.01)
Gender (ref: male)	0.60 (0.33, 1.08)	1.19 (0.50, 2.82)	0.97 (0.55, 1.71)	0.76 (0.51, 1.14)	−0.08 (−0.32, 0.16)	0.16 (−0.03, 0.34)	−0.06 (−0.22, 0.11)
Annual income (ref ≤ $25,000)	1.00	1.00	1.00	1.00	1.00	1.00	1.00
$25,001-$50,000	1.29 (0.49, 3.44)	0.97 (0.27, 3.48)	1.02 (0.40, 2.62)	1.08 (0.50, 2.12)	0.06 (−0.34, 0.46)	−0.04 (−0.35, 0.26)	−0.09 (−0.36, 0.18)
$50,001-$75,000	**3.08 (1.06, 8.98)**	0.19 (0.03, 1.18)	1.63 (0.57, 4.63)	1.17 (0.54, 2.51)	0.23 (−0.22, 0.67)	0.03 (−0.31, 0.37)	0.03 (−0.27, 0.34)
$75,001-$100,000	2.04 (0.65, 6.43)	0.80 (0.15, 4.18)	1.41 (0.47, 4.25)	1.40 (0.63, 3.11)	0.24 (−0.23, 0.72)	−0.24 (−0.60, 0.13)	−0.19 (−0.52, 0.13)
$100,000 +	1.96 (0.68, 5.64)	0.70 (0.16, 3.10)	1.09 (0.38, 3.11)	1.30 (0.62, 2.73)	0.22 (−0.22, 0.66)	−0.06 (−0.39, 0.28)	0.21 (−0.51, 0.09)
Race/Ethnicity (ref: non-Hispanic white)	1.00	1.00	1.00	1.00	1.00	1.00	1.00
African-American/Black	**2.15 (1.01, 4.58)**	1.74 (0.60, 5.10)	1.10 (0.53, 2.29)	1.40 (0.82, 2.39)	**0.42 (0.10, 0.74)**	**−0.45 (-0.69, -0.21)**	**−0.51 (-0.72, -0.29)**
Hispanic/LatinX	1.07 (0.44, 2.59)	0.84 (0.23, 3.04)	**3.62 (1.49, 8.77)**	1.58 (0.88, 2.85)	0.24 (−0.13, 0.61)	−0.28 (−0.57, 0.00)	**−0.34 (-0.59, -0.08)**
Other	2.37 (0.88, 6.35)	0.10 (0.01, 1.19)	0.94 (0.35, 2.48)	0.86 (0.43, 1.73)	−0.00 (−0.44, 0.43)	0.02 (−0.32, 0.35)	0.12 (−0.18, 0.41)
Education (ref: ≤ bachelor’s degree)	1.00	1.00	1.00	1.00	1.00	1.00	1.00
High school or less	2.27 (0.86, 5.99)	0.64 (0.15, 2.83)	0.76 (0.29, 2.00)	0.98 (0.48, 2.00)	**−0.57 (-0.99, -0.16)**	**0.55 (0.23, 0.86)**	0.26 (−0.02, 0.54)
Some college	1.04 (0.52, 2.09)	0.92 (0.30, 2.76)	0.54 (0.28, 1.08)	0.72 (0.43, 1.20)	**−0.38 (-0.67, -0.09)**	**0.27 (0.04, 0.49)**	**0.28 (0.08, 0.48)**
Graduate level degree	2.91 (0.89, 9.52)	0.18 (0.01, 2.44)	2.00 (0.62, 6.51)	1.87 (0.87, 4.01)	−0.35 (−0.85, 0.16)	0.16 (−0.23, 0.55)	0.26 (−0.08, 0.61)
Constant	**<0.01 (<0.01, 0.06)**	**0.01 (<0.01, 0.39)**	**0.51 (0.05, 5.58)**	0.46 (0.08, 2.76)	**3.08 (2.05, 4.10)**	**1.61 (0.83, 2.40)**	**2.42 (1.72, 3.13)**

Note. All variables are controlled for in overall model; CI = confidence interval; NDSS = Nicotine Dependence Syndrome Scale; Bolded values are significant at *P* < .05.

## Discussion

To our knowledge, this is the first study to create and then validate a scale assessing perceptions of nicotine addiction that comprehensively aligns with the DSM-5 dimensions of tobacco use disorder. As each item within the NAP was created using this framework, item difficulty was largely endorsed (ie, respondents indicated similar responses for each item as being indicative of nicotine addiction), suggesting that perceptions of nicotine addiction generally align with the clinical diagnosis of nicotine dependence. Using item response theory, three items assessing the factor “Consistent Desire to Quit/Reduce Use” were not effective at differentiating between individuals who perceived vaping behaviors to be more indicative of addiction vs less indicative of addiction. However, according to clinical guidelines this is an important facet of nicotine addiction. Thus, this factor was retained when establishing criterion validity. Study results also suggest that perceptions of nicotine addiction can be reliably measured in a general adult sample of people who actively use an e-cigarette and those who do not use e-cigarettes with adequate internal validity. In this regard, the 23-item NAP scale assesses perceptions of nicotine addiction as a unique construct.

In establishing convergent validity, it was anticipated that the NAP scale would converge with single item assessments of health risk perceptions used widely in research.^36^ Our study found that as NAP scores increase, so do perceptions of health harm. Higher NAP scores were related to increased perceptions of harm to one’s own health and other’s health. Additionally, perceptions that e-cigarettes are more harmful than combustible cigarettes were related to higher overall NAP scores. Interestingly, there was a small yet significant correlation between perceived likelihood of becoming addicted to e-cigarettes and NAP scores. This may be attributable to people’s underestimation of the addictiveness of e-cigarettes,^50^ or that people do not have a clear understanding of what addiction is. Without this understanding, people may be unable to estimate the likelihood of becoming addicted to nicotine. The NAP may provide deeper insight into people’s mental schemas surrounding the development of nicotine addiction.

In establishing discriminant validity, we anticipated that the NAP scale would be discernable from existing nicotine dependence measures (ie, FTND, NDSS) as the former assesses perceptions related to behaviors, and the latter assesses addictive behavior. Our findings suggest that the NAP assesses a different construct than that of the FTND and NDSS (ie, perceptions of addiction vs assessments of addiction severity). Given that the FTND and NDSS are both considered measures of addiction severity, we expected a moderate to large correlation between these two measures. However, within this sample, these scores were not related. This lends support to the concept that it is difficult to compare scores between the FTND and NDSS as they do not ask comparable questions despite aims to assess the same construct. Moreover, this exemplifies issues found in research that having non standardized measures assessing one construct may challenge public health efforts due to uncertainty or lack of applicability to the general public.^51,52^

In establishing criterion validity, we anticipated that the NAP scale would relate to vaping-related behaviors and intentions. Our findings are supported by previous research where those with higher perceptions of addiction are more likely to report intentions of quitting in the near future.^53^ However, NAP scores were significantly related to 6 month quit intentions, but not 30 day quit intentions. Research has found that smokers who are concerned about relapsing are less likely to attempt quitting,^54^ and in this regard, the current study sample of e-cigarette users may be interested in quitting in the near future, but not yet prepared to make an actual attempt in the next month. Moreover, this may also provide insight into this sample’s lack of quit attempts over the past year (56% reported zero attempts). Additionally, it was anticipated that higher NAP scores would be associated with less past 30-day e-cigarette use. Within this sample, smokers with higher NAP scores were likely to report using e-cigarettes on more days out of the past month. In line with existing qualitative research, this sample may feel less control over their vaping and thus, use these products daily to satiate their craving/symptoms of addiction.^55^

Treating addiction can be difficult, in part due to people’s inability to acknowledge they are addicted, or their desire to not be labeled an addict due to the negative connotation surrounding this term.^56^ Much of the work educating the public has prioritized the *consequences* of nicotine addiction rather than the *experiences* of nicotine addiction. This may help explain why lay people’s perceptions of nicotine addiction encompass only five of 11 clinical dimensions. Education efforts should emphasize the dimensions of addiction –rather than a simple definition – so that people can recognize that they, or a loved one, is addicted and seek help prior to experiencing particularly damaging consequences of addiction (eg, lung cancer, COPD, death, etc.).^57^

Most current preventative and intervention programs highlight the negative consequences of tobacco use, but the lack of emphasis surrounding other dimensions of addiction (eg, persistent desire to reduce/control use, recreational activities are given up/reduced due to substance use, etc.) may be diminishing the fact that active smokers are using tobacco against their will. This last part, using tobacco against one’s will, is a clinically recognized aspect of addiction and is demonstrated epidemiologically that over 70% of current smokers regret initiating in the first place.^58^ Yet, this aspect of addiction is not well understood by the general public. Future research may choose to examine whether people’s knowledge in this area could be used to help prevent future tobacco use.

### Limitations/Future Directions

A limitation of the study is the time period in which data were collected (late December 2023). Nearing the end of the year, some individuals make resolutions for the upcoming new year, predominantly related to changing health behavior.^59^ In this regard, respondents may have been more inclined to report having future intentions to quit vaping. However, among the current study sample, almost half of active e-cigarette users reported no intention of quitting vaping (49.27%). Data were collected at one time point and were therefore, cross-sectional. Future studies will be needed to assess how people’s perceptions of nicotine addiction changes across time. This may also be helpful in understanding whether and/or how perceptions change and ultimately, their impact on behavior change. Additionally, future research should employ more advanced statistical techniques (eg, structural equation modeling) with the aim of further examining the relationships between perceptions of nicotine addiction and smoking behaviors. Some latent factors may be more relevant to cessation outcomes than an overall scale score. Lastly, we recognize that a 23-item scale may contribute to participant response burden, and that some items are highly correlated with one another (see Supplemental Material 4). In this regard, we plan on improving the NAP scale by creating short-form versions that remove highly correlated items but still encompass as many DSM-5 dimensions as possible and assess their reliability and validity among different samples.

### Conclusions

To our knowledge, this is the first study to create a comprehensive scale assessing perceptions of nicotine addiction using the clinical dimensions of tobacco use disorder as a framework. Items in the final NAP scale returned acceptable diagnostics using IRT guidelines and construct validity was established using convergent, discriminant, and criterion validity. The findings from this study suggest that for most, perceptions of nicotine addiction may not align with DSM-5 clinical criteria, which may be due potentially to the lack of education surrounding addiction. Future research examining perceptions of nicotine addiction can utilize the NAP scale to better understand lay people’s understanding of addiction and its relationship to vaping related behaviors. Public health efforts can utilize the scale to adapt curriculum to focus on the experiences of nicotine addiction, rather than just the consequences of nicotine addiction to promote healthy behavior.

## Supplemental Material

Supplemental Material - Assessing Perceptions and Behaviors Related to Vaping Nicotine: The Nicotine Addiction Perceptions Scale Supplemental Material for Assessing Perceptions and Behaviors Related to Vaping Nicotine: The Nicotine Addiction Perceptions Scale by Allison A. Temourian, Anna V. Song, and Anna E. Epperson in Tobacco Use Insights

## Data Availability

Data and analysis code can be made available upon reasonable request to the corresponding author. The full survey can be found in Supplemental Material 6.
