# Supplementary material for: Assessing Perceptions and Behaviors Related to Vaping Nicotine: The Nicotine Addiction Perceptions Scale
Source: Tob Use Insights. 2025 Apr 26;18:1179173X251336468. doi: 10.1177/1179173X251336468 (PMC12035213; doi:10.1177/1179173X251336468)
Supplement: Supplemental Material - Assessing Perceptions and Behaviors Related to Vaping Nicotine: The Nicotine Addiction Perceptions Scale [file sj-pdf-1-tui-10.1177_1179173X251336468.pdf]

Supplemental Material 1
36-item NAP Scale
Below we are going to give you a list of behaviors related to ***nicotine based vapes/e-cigarettes****.*
On a scale from 1-5, how important are any of these behaviors in telling you a person is addicted to ***vapes/e-cigarettes****?* (1 = not at all important, 5 = extremely important)

1. Vaping more now than when they first started
2. Taking more puffs/hits throughout the day
3. Taking more time per day to vape
4. Having difficulty quitting vaping
5. Having difficulty reducing their vaping
6. Often thinking about quitting vaping
7. Often thinking about reducing their vaping
8. Constantly wanting to quit vaping
9. Spending a lot of time thinking about the next time they can vape
10. Most thoughts are related to how they can vape again
11. Craving a vape when they are not smoking
12. Vaping gets in the way of their daily life
13. Vaping negatively impacts their work productivity
14. Arguing with other people over their vaping
15. Social life is negatively impacted by their vaping
16. Vaping causes problems with their social circle
17. Person gives up their social life in order to vape
18. Giving up part of their job because of vaping
19. Giving up hobbies because of vaping
20. Cutting down on hobbies because of vaping
21. Vaping in places that might cause a fire
22. Continued vaping even though they are aware it is bad for them
23. Continued vaping even after experiencing negative health effects from vaping
24. Continued vaping even though it causes conflict in their familial relationships
25. Continued vaping even though it causes conflict in their relationships with friends
26. Continued vaping even though it causes conflict in their romantic relationships
27. Needing to vape more to get the same buzz
28. Becoming irritable when they do not vape
29. Being on edge when they do not vape
30. Having a hard time concentrating when they do not vape
31. Having trouble sleeping when they do not vape
32. Feeling anxious when they do not vape
33. Feeling sad when they do not vape
34. Eating more when they do not vape
35. Drinking more alcoholic beverages when they do not vape
36. Drinking less alcoholic beverages when they do not vape

Supplemental Material 2

Prolific Sample Descriptive Statistics (%)

	Non-Vapers (n = 275)	Vapers (n= 274)	Total Sample (n = 549)
**Gender**			
Female	49.09	43.07	46.08
Male	47.27	55.47	51.37
Other	3.64	1.46	2.55
**Age (Mean, SD)**	38.68 (13.49)	40.44 (11.72)	39.56 (12.66)
**Race/Ethnicity**			
African-American/Black	8.36	21.17	14.75
Hispanic	10.18	13.14	11.66
Other	13.09	9.12	11.11
White Non-Hispanic	68.36	56.57	62.48
**Education Level**			
High school or lower	12.36	12.77	12.57
Some college or Associate's degree	29.45	38.69	34.06
Bachelor's degree	38.55	41.24	39.89
Graduate or Professional degree	19.64	7.30	13.48
**Annual Income**			
Up to $10,000	3.64	2.92	3.28
$11,000 - $25,000	10.55	12.41	11.48
$25,001 - $50,000	22.55	22.63	22.59
$50,001 - $75,000	20.73	18.98	19.85
$75,001 - $100,000	16.36	13.87	15.12
$100,001 - $200,000	18.18	27.37	22.77
Over $200,000	7.64	1.82	4.74
**Current cigarette user**	8.00	47.84	34.62
**Primary reason for using e-cigarettes**			
To quit smoking	7.26	25.18	19.60
To cut down smoking	3.23	20.07	14.82
To use when cannot or are not allowed to smoke	1.61	8.03	6.03
Enjoyment	2.42	41.61	29.40
Curiosity	83.06	3.65	28.39
Other	2.42	1.46	1.76
**E-cigarette Initiation Intentions**			
No intention	97.09	--	--
Within next 6 months, but not 30 days	0.73	--	--
Within next 30 days	2.18	--	--
**E-cigarette Cessation Intentions**			
No intention	--	49.27	--
Within next 6 months, but not 30 days	--	13.14	--
Within next 30 days	--	37.59	--
**E-cigarette Composition**			
Nicotine	64.71	82.12	76.84

Mint or Menthol flavor	28.57	51.82	44.78
Some other flavor	65.55	67.52	66.92
CBD	26.05	23.72	24.37
THC	36.97	30.29	32.32
Own mix	0.84	10.95	7.89
**Daily E-cigarette Use**			
1-9 times per day	--	46.52	--
10-24 times per day	--	28.21	--
25+ times per day	--	25.27	--
**Monthly E-cigarette Use**			
2-10 days per month	--	18.98	--
11-24 days per month	--	20.80	--
25 + days per month	--	60.22	--

Note. SD = standard deviation; Non-Vapers = may have used an e-cigarette/vape no more than 20 times in their life (per Prolific definition); Vapers = active (past 30-day) use of e-cigarette/vaping device.

Supplemental Material 3. NAP Scale Exploratory Factor Analysis (EFA) Findings

Table 1. EFA Sample Descriptive Statistics (%)

	Current Non-Vapers (n = 154)	Current Vapers (n= 25)	Total Sample (n = 179)
**Gender**			
Female			79.78
Male			16.85
Other			3.37
**Age (Mean, SD)**			20.53 (2.69)
**Race/Ethnicity**			
Asian American			21.23
Hispanic/Latinx			65.17
White Non-Hispanic			10.11
Other			3.49
**Annual Income**			
Up to $10,000			12.36
$11,000 - $25,000			12.36
$25,001 - $50,000			17.98
$50,001 - $75,000			11.24
$75,001 - $100,000			10.11
$100,001 - $200,000			8.43
Over $200,000			1.69
Unsure			25.84
**Parent owns home**			52.25
**Ever e-cigarette user**			44.13
**Current cigarette user**			1.68
**Primary reason for using e-cigarettes**			
To quit smoking			8.00
To cut down smoking			--
To use when cannot or are not allowed to smoke			8.00
Enjoyment			28.00
Curiosity			48.00
Other			8.00
**E-cigarette Initiation Intentions**			
No intention	99.35	--	--
Within next 6 months, but not 30 days	0.65	--	--
Within next 30 days	--	--	--
**E-cigarette Cessation Intentions**			
No intention	--	40.00	--
Within next 6 months, but not 30 days	--	32.00	--
Within next 30 days	--	28.00	--
**E-cigarette Composition**			
Nicotine	--	76.00	--

Mint or Menthol flavor	--	31.82	--
Some other flavor	--	59.09	--
CBD	--	33.33	--
THC	--	52.17	--
Own mix	--	--	--
**Daily E-cigarette Use**			
1-9 times per day	--	61.90	--
10-24 times per day	--	23.81	--
25+ times per day	--	14.29	--
**Monthly E-cigarette Use**			
1-10 days per month	--	62.50	--
11-24 days per month	--	16.67	--
25 + days per month	--	20.83	--

Note. SD = standard deviation

Table 2. EFA related findings

**Test**	**Test Statistic**
Mardia's test of multivariate normality	$p < 0.001$
Kaiser-Meyer-Olkin Measure of Sampling Adequacy	0.94
Bartlett's Test of Sphericity	$\chi 2 = 6181.11$, df = 630, $p = 0.000$

Note. N = 177; Missing responses were verified to be missing completely at random ($p = 0.991$) and were omitted from analysis (n = 2).

Table 3. EFA Factor Variance

**Factor**	**% of total variance**
1: Continued use despite harm	21%
2: Withdrawal	15%
3: Tolerance	14%
4: Social impact	13%
5: Desire to quit/reduce use	12%
6: Substitution to other behavior	9%

Note. N = 177; Principal axis factoring extraction method was used to determine the appropriate number of factors to extract; Oblique rotation method (oblimin) was used to allow for correlations between factors; estimated RMSEA = 0.088; estimated Tucker Lewis Index = 0.926.

Supplemental Material 4

Item Correlations of 23-item NAP scale

NAP	1	2	3	6	7	8	11	12	13	17	18	19	21	22	23	24	25	26	27	28	29	30	31
**1**	1																						
**2**	.87	1																					
**3**	.81	.83	1																				
**6**	.28	.29	.31	1																			
**7**	.28	.26	.31	.85	1																		
**8**	.22	.27	.28	.72	.77	1																	
**11**	.63	.66	.64	.30	.27	.30	1																
**12**	.57	.60	.63	.29	.27	.44	.72	1															
**13**	.48	.53	.57	.33	.28	.40	.65	.85	1														
**17**	.53	.56	.62	.27	.23	.38	.68	.85	.8	1													
**18**	.50	.56	.57	.25	.20	.39	.66	.86	.82	.90	1												
**19**	.47	.50	.57	.28	.25	.41	.61	.82	.72	.83	.87	1											
**21**	.34	.38	.41	.28	.27	.32	.46	.52	.54	.53	.56	.46	1										
**22**	.54	.54	.57	.35	.35	.36	.58	.49	.51	.49	.47	.46	.55	1									
**23**	.56	.55	.58	.31	.28	.37	.61	.68	.65	.67	.65	.58	.57	.75	1								
**24**	.47	.48	.55	.27	.25	.30	.58	.67	.69	.71	.68	.63	.56	.66	.73	1							
**25**	.50	.48	.53	.26	.23	.30	.56	.65	.67	.74	.70	.68	.53	.64	.66	.88	1						
**26**	.53	.55	.57	.28	.24	.31	.60	.71	.67	.75	.74	.69	.53	.63	.69	.84	.91	1					
**27**	.54	.60	.59	.24	.23	.25	.66	.61	.58	.62	.60	.56	.45	.60	.63	.63	.61	.62	1				
**28**	.57	.58	.60	.25	.26	.33	.73	.69	.66	.65	.66	.59	.53	.61	.69	.63	.62	.65	.80	1			
**29**	.56	.56	.58	.26	.24	.31	.73	.68	.66	.66	.64	.59	.50	.63	.69	.59	.61	.65	.75	.92	1		
**30**	.53	.56	.59	.34	.30	.35	.74	.69	.70	.72	.71	.66	.52	.61	.70	.63	.65	.69	.71	.83	.87	1	
**31**	.49	.52	.56	.30	.28	.30	.65	.66	.65	.65	.68	.66	.56	.61	.67	.66	.65	.67	.68	.82	.84	.87	1

Supplemental Material 5. Full Regression Results (including covariates)

Six month quit intentions. For every unit increase in perceptions of nicotine addiction, intentions to quit using e-cigarettes in the next six months increased (OR = 1.03, 95% CI: 1.01, 1.04; p = 0.007; Table 15). For each unit of increased perception of comparative e-cigarette harm, intentions to quit using e-cigarettes in the next six months increased (OR = 1.47, 95% CI: 1.05, 2.06; p = 0.026). Among those making between $50,001- $75,000 annually, intentions to quit using e-cigarettes in the next six months increased (OR = 3.08, 95% CI: 1.06, 8.98, p = 0.039) compared to those making less than $20,000 annually. Compared to non-Hispanic white adults, African American/Black adults were twice as likely to report intentions of quitting using e-cigarettes in the next six months (OR = 2.14, 95% CI: 1.01, 4.58, p = 0.048).

Thirty day quit intentions. For each unit of increased perception of comparative e-cigarette harm, desire to quit using e-cigarettes in the next 30 days increased (OR = 1.89, 95% CI: 1.16, 3.08; p = 0.010). Compared to those using e-cigarettes for enjoyment, those using e-cigarettes to cut down on smoking (OR = 3.70, 95% CI: 1.16, 11.75; p = 0.027), and curiosity reported desire to quit using e-cigarettes in the next 30 days (OR = 25.09, 95% CI: 3.57, 176.30; p = 0.001).

Attempted cessation in past 12 months. For each unit of increased perception of comparative e-cigarette harm, the likelihood of having tried to quit using e-cigarettes in the past 12 months increased (OR = 1.57, 95% CI: 1.12, 2.20; p = 0.009; Table X. Logistic Regression Results). Compared to those using e-cigarettes for enjoyment, those using e-cigarettes to use when they cannot or are not allowed to smoke were three times as likely to report having tried to quit using e-cigarettes in the past 12 months (OR = 3.19, 95% CI: 1.11, 9.18; p = 0.032). Compared to non-Hispanic White e-cigarette users, Hispanic/Latinx e-cigarette users were three times as likely to report having tried quit using e-cigarettes in the past 12 months (OR = 3.62, 95% CI: 1.49, 8.77, p = 0.004).

Number of quit attempts in past 12 months. For each unit of increased perception of nicotine addiction, the number of quit attempts over the past 12 months decreased by a factor of 0.99 (95% CI [0.98, 1.00]; Table 16). For each unit of increased perception of comparative e-cigarette harm, the number of quit attempts over the past 12 months increased by a factor of 1.62 (95 % CI [1.28, 2.05]). For every unit increase in nicotine dependence, the number of quit attempts over the past 12 months increased by a factor of 1.08 (95% CI [1.03, 1.14]). Compared to those using e-cigarettes for enjoyment, those using e-cigarettes to quit smoking reported increased number of quit attempts over the past 12 months by a factor of 2.00 (95% CI [1.17, 3.40]). For every unit increase in age, the number of quit attempts over the past 12 months decreased by a factor of 0.98 (95% CI [0.96, 1.00]). For every unit increase in mental distress, the number of quit attempts over the past 12 months increased by a factor of 1.24 (95% CI [1.01, 1.53]).

Measured time to first use of e-cigarette after waking. Compared to those using e-cigarettes for enjoyment, those using e-cigarettes to quit smoking were likely to use their first e-cigarette sooner upon waking (b = -0.67, p < 0.001; Table 17). Compared to those who use e-liquid that does not contain nicotine, those who use e-liquid that contains nicotine were likely to use their first e-cigarette sooner upon waking (b = -0.49, p = 0.003). Compared to non-Hispanic White e-cigarette users, African American/Black e-cigarette users (b = 0.42, p = 0.010) were likely to use their first e-cigarette later upon waking. Compared to those with a bachelor's degree, those with a high school education (b = -0.57, p = 0.007) and those with some college education (b = -0.38, p = 0.011) were likely to use their first e-cigarette sooner upon waking.

Daily e-cigarette use. Compared to those using e-cigarettes for enjoyment, those using e-cigarettes to quit smoking cigarettes were likely to take more e-cigarette puffs per day (b = 0.28, $p = 0.026$; Table X: Linear Regression Results). Compared to non-Hispanic White e-cigarette users, African American/Black e-cigarette users were likely to take less e-cigarette puffs per day (b = -0.45, $p < 0.001$). Compared to those with a bachelor's degree, those with a high school education (b = 0.55, $p = 0.001$) and those with some college education (b = 0.27, $p = 0.019$) were likely to take more e-cigarette puffs per day.

Past 30-day e-cigarette use. For every unit increase in perceptions of nicotine addiction, the number of days vaped in the past 30 days increased (b = 0.00, $p = 0.039$). For every unit increase in comparative harm, the number of days vaped in the past 30 days decreased (b = -0.15, $p = 0.003$). Compared to those who use e-liquid that does not contain nicotine, those who use e-liquid that contains nicotine were likely to vape on more days out of the past 30 days (b = 0.50, $p < 0.001$). Compared to those using e-cigarettes for enjoyment, those using e-cigarettes to quit smoking were likely to vape on more days out of the past 30 days (b = 0.25, $p = 0.026$). For every unit increase in mental distress, the number of days vaped in the past 30 days decreased (b = -0.09, $p = 0.033$). Compared to non-Hispanic White e-cigarette users, African American/Black (b = -0.51, $p < 0.001$) and Hispanic/Latinx e-cigarette users (b = -0.34, $p = 0.010$) were likely to vape on less days out of the past 30 days. Compared to those with a bachelor's degree, those with some college education were likely to vape on more days out of the past 30 days (b = 0.28, $p = 0.006$).

Supplemental Material 6
Survey Questionnaire

This first set of questions regards your **e-cigarette/vape** use (see below for visual representation). Please note that the terms **e-cigarette** and **vape/vaping** are used interchangeably. Your answers are extremely important to us, so please answer each question as honestly as you can. If you can't remember or aren't sure, just answer the best you can. There are no right or wrong answers. Your identity is not linked to this survey and we will not be able to link your name to your responses.

1. Have you ever used an **e-cigarette** or **vape**?

   O Yes (1)

   O No (0)

2. Have you used an **e-cigarette** in the **past 12 months**?

   O Yes (1)

   O No (0)

3. Have you used an **e-cigarette** in the **past 30 days?**

   O Yes (1)

   O No (0)

4. How many **times in a month** do you currently use an **e-cigarette** device? (click below for pull-down menu and choose a number from 0-30 days)

▼ 0 days (0) ... 30 days (30)

5. On average, how many **times a day** do you use **e-cigarettes**?

▼ 0 (0) ... 30 or more times a day (30)

6. How soon after waking do you smoke your first **e-cigarette**?

O Within 5 minutes (1)

O 5-30 minutes (2)

O 31-60 minutes (3)

O Longer than 60 minutes (4)

7. Do you feel like you are addicted to **e-cigarettes**?

O Yes (1)

O No (0)

8. When you smoked an **e-cigarette** in the **past 30 days,** did it contain any of the following? (Select all that apply)

	Yes, my vape contained this (1)	No, my vape did not contain this (0)	I'm not sure (3)
8. Nicotine	O	O	O
9. Mint or Menthol flavor	O	O	O
10. Some other flavor	O	O	O
11. CBD	O	O	O
12. THC	O	O	O
13. Your own mix of e-liquid	O	O	O

14. Please indicate the brand of **e-cigarettes** you have used in the past (select all that apply)

- [ ] BLVK (1)
- [ ] Elf Bar (2)
- [ ] FLUM (3)
- [ ] Fume (4)
- [ ] Hyde (5)
- [ ] Juul (6)
- [ ] Posh (7)
- [ ] Puff bar (8)
- [ ] Vuse (9)
- [ ] ZOOVOO (DRAGBAR) (10)
- [ ] Other (11) ____
- [ ] I'm not sure (12)

15. What is your primary reason for using an **e-cigarette** device?

- O To quit smoking (1)
- O To cut down smoking (2)
- O To use when I cannot or am not allowed to smoke (3)
- O Because I enjoy it (4)
- O Curiosity/ just wanted to try them (5)
- O Some other reason (6)

16. Have you ever tried to quit using **e-cigarettes**, but couldn't?

- O Yes (1)
- O No (0)

17. In the past year, did you quit **vaping** voluntarily for at least 24 hours?

- O Yes (1)
- O No (0)

18. In the past year, how many times did you try to quit **vaping/e-cigarettes**?

▼ 0 (0) ... 12+ (12)

19. Do you have serious thoughts about quitting **vaping/using e-cigarettes?**

- O Yes, within the next 30 days (1)
- O Yes, within the next 6 months, but not in the next 30 days (2)
- O No, I am not thinking of quitting within the next 6 months (0)

20. Have you ever spoken to a doctor or pharmacist about wanting to quit using **e-cigarettes**?

- O Yes, a doctor (1)
- O Yes, a pharmacist (2)
- O Yes, both a doctor and a pharmacist (3)
- O No (0)

Please indicate how well each of the following statements describes you.

	Not at all true (1)	Somewhat true (2)	Moderately true (3)	Very true (4)	Extremely true (5)
21. After not vaping for a while, I need to vape to relieve feelings of restlessness and irritability	O	O	O	O	O
22. Whenever I go without vaping for a few hours, I experience craving	O	O	O	O	O
23. After not vaping for a while, I need to vape in order to keep myself from experiencing any discomfort	O	O	O	O	O
24. When I am really craving a vape, it feels like I'm in the grip of some unknown force that I cannot control	O	O	O	O	O
25. I feel a sense of control over my vaping. I can "take it or leave it" at any time	O	O	O	O	O
26. I tend to avoid restaurants that don't allow vaping, even if I would otherwise enjoy the food	O	O	O	O	O
27. Sometimes I decline offers to visit with my non-smoking friends because I know I'll feel uncomfortable if I vape	O	O	O	O	O
28. Even if traveling a long distance, I'd rather not travel by airplane because I wouldn't be allowed to vape	O	O	O	O	O
29. Since the time when I became a regular vaper, the amount I vape has either stayed the same or has decreased somewhat	O	O	O	O	O

	Not at all true (1)	Somewhat true (2)	Moderately true (3)	Very true (4)	Extremely true (5)
30. Compared to when I first started vaping, I need to vape a lot more now in order to get what I really want out of it	O	O	O	O	O
31. Compared to when I first started vaping, I can vape much, much more now before I start to feel nauseated or ill	O	O	O	O	O
32. It's hard to estimate how many puffs I vape per day because the number often changes	O	O	O	O	O
33. My vaping pattern is very irregular throughout the day. It is not unusual for me to take many puffs in an hour, then not have another puff until hours later	O	O	O	O	O
34. The number of puffs I take per day is often influenced by other factors - how I'm feeling, what I'm doing, etc.	O	O	O	O	O
35. I vape at different rates in different situations	O	O	O	O	O
36. My vaping is not much affected by other things. I vape about the same amount whether I'm relaxed or working, happy or sad, alone or with others, etc.	O	O	O	O	O
37. My vaping is fairly regular throughout the day	O	O	O	O	O
38. I vape consistently and regularly throughout the day	O	O	O	O	O
39. I vape about the same amount on weekends as on weekdays	O	O	O	O	O

40. Do you find it difficult to refrain from vaping in places where it is forbidden (e.g., in church, at the library, in the cinema)?

- O Yes (1)
- O No (0)

41. Which vaping session would you hate most to give up?

- O The first one in the morning (1)
- O During or after meals (2)
- O During or after stressful situations (3)
- O None of the above (4)

42. Do you vape more frequently during the first hours after waking than during the rest of the day?

- O Yes (1)
- O No (0)

43. Do you vape when you are so ill that you are in bed most of the day?

- O Yes (1)
- O No (0)

If you continue to use e-cigarettes at your current rate, how likely is it that you will...

	Very unlikely (1)	Unlikely (2)	Neither likely nor unlikely (3)	Likely (4)	Very likely (5)
44. Become addicted	O	O	O	O	O
45. Harm your own health	O	O	O	O	O
46. Harm someone else's health with second hand vape smoke	O	O	O	O	O

47. Compared with cigarettes, how harmful are e-cigarettes to a person's health?

- O Much less harmful than cigarettes (1)
- O Somewhat less harmful than cigarettes (2)
- O Equally harmful as cigarettes (3)
- O Somewhat more harmful than cigarettes (4)
- O Much more harmful than cigarettes (5)

48. If you had to describe **nicotine**, how confident would you be in your description?

- O Not confident at all (1)
- O Slightly confident (2)
- O Somewhat confident (3)
- O Fairly confident (4)
- O Completely confident (5)

49. True or False. Nicotine is a highly addictive substance that is found naturally in tobacco leaves and can be produced synthetically in a lab.

- O True (1)
- O False (0)

Below we are going to give you a list of behaviors related to **nicotine based vapes/e-cigarettes**. On a scale from 1-5, how important are any of these behaviors in telling you a person is addicted to **vapes/e-cigarettes**?

	Not at all important (1)	Slightly importan t (2)	Moderately important (3)	Very important (4)	Extremely important (5)
50. Vaping more now than when they first started	O	O	O	O	O
51. Taking more puffs/hits throughout the day	O	O	O	O	O
52. Taking more time per day to vape	O	O	O	O	O
53. Having difficulty quitting vaping	O	O	O	O	O
54. Having difficulty reducing their vaping	O	O	O	O	O
55. Often thinking about quitting vaping	O	O	O	O	O
56. Often thinking about reducing their vaping	O	O	O	O	O
57. Constantly wanting to quit vaping	O	O	O	O	O
58. Spending a lot of time thinking about the next time they can vape	O	O	O	O	O
59. Most thoughts are related to how they can vape again	O	O	O	O	O
60. Craving a vape when they are not vaping	O	O	O	O	O
61. Vaping gets in the way of their daily life	O	O	O	O	O
62. Vaping negatively impacts their work productivity	O	O	O	O	O
63. Arguing with other people over their vaping	O	O	O	O	O
64. Social life is negatively impacted by their vaping	O	O	O	O	O

65. Vaping causes problems with their social circle	O	O	O	O	O

	Not at all important (1)	Slightly importan t (2)	Moderately important (3)	Very important (4)	Extremely important (5)
66. Person gives up their social life in order to vape	O	O	O	O	O
67. Giving up part of their job because of vaping	O	O	O	O	O
68. Giving up hobbies because of vaping	O	O	O	O	O
69. Cutting down on hobbies because of vaping	O	O	O	O	O
70. Vaping in places that might cause a fire	O	O	O	O	O
71. Continued vaping even though they are aware it is bad for them	O	O	O	O	O
72. Continued vaping even after experiencing negative health effects from vaping	O	O	O	O	O
73. Continued vaping even though it causes conflict in their familial relationships	O	O	O	O	O
74. Continued vaping even though it causes conflict in their relationships with friends	O	O	O	O	O
75. Continued vaping even though it causes conflict in their romantic relationships	O	O	O	O	O
76. Needing to vape more to get the same buzz	O	O	O	O	O
77. Becoming irritable when they do not vape	O	O	O	O	O
78. Being on edge when they do not vape	O	O	O	O	O
79. Having a hard time concentrating when they do not vape	O	O	O	O	O
80. Having trouble sleeping when they	O	O	O	O	O

81. Feeling anxious when they do not vape	O	O	O	O	O
82. Feeling sad when they do not vape	O	O	O	O	O
83. Eating more when they do not vape	O	O	O	O	O
84. Drinking more alcoholic beverages when they do not vape	O	O	O	O	O
85. Drinking less alcoholic beverages when they do not vape	O	O	O	O	O

This next set of questions regards your **cigarette** use. Your answers are extremely important to us, so please answer each question as honestly as you can. If you can't remember or aren't sure, just answer the best you can. There are no right or wrong answers. Your identity is not linked to this survey and we will not be able to link your name to your responses.

86. Have you ever smoked a **cigarette** (even one puff)?

O Yes (1)

O No (0)

87. Have you smoked a **cigarette** in the past **30 days?**

O Yes (1)

O No (0)

88. Have you smoked a **cigarette** in the past **12 months**?

O Yes (1)

O No (0)

89. Are you thinking of using **cigarettes** in the near future?

- O Yes, within the next 30 days (1)
- O Yes, within the next 6 months, but not the next 30 days (2)
- O No, I am not thinking of using cigarettes within the next 6 months (0)

90. On how many of the past **30 days** did you smoke **cigarettes**?(please choose a number from 0-30 from the pull-down menu)

▼ 0 (0) ... 30 (30)

91. On average, how many **cigarettes a day** do you smoke? (Please note: 1 Pack = 20 cigarettes)

▼ 0 (0) ... 100 or more (100)

92. How soon after waking do you smoke your first **cigarette**?

- O Within 5 minutes (1)
- O 6-30 minutes (2)
- O 31-60 minutes (3)
- O After 60 minutes (4)
- O This question is not clear (5)

93. Do you feel like you are addicted to **cigarettes**?

- O Yes (1)
- O No (0)

94. For how many years have you smoked?

____

95. Have you ever tried to quit smoking **cigarettes**, but couldn't?

- O Yes (1)
- O No (0)

96. In the past year, did you quit smoking voluntarily for at least 24 hours?

- O Yes (1)
- O No (0)

97. In the past year, how many times did you try to quit smoking **cigarettes**?

▼ 0 (0) ... 12+ (12)

98. Do you have serious thoughts about quitting smoking **cigarettes?**

- Yes, within the next 30 days (1)
- Yes, within the next 6 months, but not in the next 30 days (2)
- No, I am not thinking of quitting within the next 6 months (0)

99. Have you ever spoken to a doctor or pharmacist about wanting to quit smoking **cigarettes**?

- Yes, a doctor (1)
- Yes, a pharmacist (2)
- Yes, both a doctor and a pharmacist (3)
- No (0)

100. Which of the following methods to quit smoking **cigarettes** have you used in the past? (Select all that you used to try to quit)

- [ ] Nicotine patch (1)
- [ ] Nicotine gum (2)
- [ ] Nicotine inhalers (3)
- [ ] Nicotine lozenge (4)
- [ ] A smokeless tobacco product (5)
- [ ] Anti-depressants (like Prozac or Zoloft or Zyban) (6)
- [ ] Chantix (7)
- [ ] Counseling/therapy (8)
- [ ] E-cigarettes (9)

☐ Marijuana products (10)

☐ Called a Quit Hotline (11)

☐ Other (12)

☐ Did not use anything (cold turkey) (13)

☐ I have never tried to quit smoking (14)

What best describes the **marijuana** products that you used to try to quit smoking **cigarettes**?

	Yes (1)	No (0)	I'm not sure (3)
101. A Joint	O	O	O
102. A Blunt	O	O	O
103. A Dab	O	O	O
104. A Vape or E-cigarette with marijuana	O	O	O
105. Marijuana edibles	O	O	O
106. Marijuana topicals or creams	O	O	O
107. Other marijuana product	O	O	O

108. To the best of your knowledge, what best describes the **THC** content of your **marijuana** product that you used to try to quit smoking **cigarettes**?

- High THC (1)
- Low THC (2)
- No THC (3)
- I'm not sure (4)
- I don't know what THC is (5)

109. To the best of your knowledge, what best describes the **CBD** content of your **marijuana** product that you used to try to quit smoking **cigarettes**?

- High CBD (1)
- Low CBD (2)
- No CBD (3)
- I'm not sure (4)
- I don't know what CBD is (5)

When you used an **e-cigarette** to try to quit smoking **cigarettes**, did it contain:

	Yes, my vape contained this (1)	No, my vape did not contain this (0)	I'm not sure (3)
110. Nicotine	o	o	o
111. Mint or Menthol flavor	o	o	o
112. Some other flavor	o	o	o
113. CBD	o	o	o
114. THC	o	o	o
115. Your own mix of e-liquid	o	o	o

Please indicate how well each of the following statements describes you.

	Not at all true (1)	Somewhat true (2)	Moderately true (3)	Very true (4)	Extremely true (5)
116. After not smoking for a while, I need to smoke to relieve feelings of restlessness and irritability	O	O	O	O	O
117. Whenever I go without a smoke for a few hours, I experience craving	O	O	O	O	O
118. After not smoking for a while, I need to smoke in order to keep myself from experiencing any discomfort	O	O	O	O	O
119. When I am really craving a cigarette, it feels like I'm in the grip of some unknown force that I cannot control	O	O	O	O	O
120. I feel a sense of control over my smoking. I can "take it or leave it" at any time	O	O	O	O	O
121. I tend to avoid restaurants that don't allow smoking, even if I would otherwise enjoy the food	O	O	O	O	O
122. Sometimes I decline offers to visit with my non-smoking friends because I know I'll feel uncomfortable if I smoke	O	O	O	O	O
123. Even if traveling a long distance, I'd rather not travel by airplane because I wouldn't be allowed to smoke	O	O	O	O	O
124. Since the time when I became a regular smoker, the amount I smoke has either stayed the same or has decreased somewhat	O	O	O	O	O

	Not at all true (1)	Somewhat true (2)	Moderately true (3)	Very true (4)	Extremely true (5)
125. Compared to when I first started smoking, I need to smoke a lot more now in order to get what I really want out of it	O	O	O	O	O
126. Compared to when I first started smoking, I can smoke much, much more now before I start to feel nauseated or ill	O	O	O	O	O
127. It's hard to estimate how many cigarettes I smoke per day because the number often changes	O	O	O	O	O
128. My smoking pattern is very irregular throughout the day. It is not unusual for me to smoke many cigarettes in an hour, then not have another one until hours later	O	O	O	O	O
129. The number of cigarettes I smoke per day is often influenced by other factors - how I'm feeling, what I'm doing, etc.	O	O	O	O	O
130. I smoke at different rates in different situations	O	O	O	O	O
131. My smoking is not much affected by other things. I smoke about the same amount whether I'm relaxed or working, happy or sad, alone or with others, etc.	O	O	O	O	O
132. My cigarette smoking is fairly regular throughout the day	O	O	O	O	O
133. I smoke consistently and regularly throughout the day	O	O	O	O	O
134. I smoke about the same amount on weekends as on weekdays	O	O	O	O	O

135. Do you find it difficult to refrain from smoking in places where it is forbidden (e.g., in church, at the library, in the cinema)?

- Yes (1)
- No (0)

136. Which smoking session would you hate most to give up?

- The first one in the morning (1)
- During or after meals (2)
- During or after stressful situations (3)
- None of the above (4)

137. Do you smoke more frequently during the first hours after waking than during the rest of the day?

- Yes (1)
- No (0)

138. Do you smoke when you are so ill that you are in bed most of the day?

- Yes (1)
- No (0)

139. Have you ever used **Marijuana?**

- Yes (1)
- No (0)

140. Have you used **Marijuana** in the **past 30 days?**

- Yes (1)
- No (0)

141. How many times in a month do you currently use **Marijuana**? (click below for pull-down menu and choose a number from 0-30 days)

▼ 0 days (0) ... 30 days or more (30)

142. On average, how many times do you smoke or consume **marijuana** products per day?

- Once a day (1)
- More than once a day (2)

143. On days when you use a **marijuana or cannabis** product**,** how soon after waking do you use that product?

- Within 5 minutes (1)
- 5-30 minutes (2)
- 31-60 minutes (3)
- Longer than 60 minutes (4)

144. Do you feel like you are addicted to **marijuana?**

- Yes (1)
- No (0)

145. In the past 30 days, what kind of **marijuana** products did you use? Select all that apply:

- ☐ Joints (1)
- ☐ Blunts (2)
- ☐ Dabs (3)
- ☐ Marijuana Vapes (4)
- ☐ Marijuana Edibles (5)
- ☐ Marijuana topicals or creams (6)
- ☐ Other product (7)

In the past **30 days**, which **marijuana** product did you use most often?

	Used least often						Used most often			
	1	2	3	4	5	6	7	8	9	10
146. Joints										
147. Blunts										
148. Dabs										
149. Marijuana Vapes										
150. Marijuana Edibles										
151. Marijuana topicals or creams										
152. Other Product										

153. To the best of your knowledge, what best describes the **THC** content of the **marijuana** products you use most often?

- High THC (1)
- Low THC (2)
- No THC (3)
- I'm not sure (4)
- I don't know what THC is (5)

154. To the best of your knowledge, what best describes the **CBD** content of the **marijuana** products you use most often?

- High CBD (1)
- Low CBD (2)
- No CBD (3)
- I'm not sure (4)
- I don't know what CBD is (5)

155. What is your primary reason for using **marijuana?**

- Medical reasons (1)
- Personal enjoyment (2)
- Curious/Just wanted to try it (3)
- To help quit smoking cigarettes (4)
- To reduce the number of cigarettes you smoke (5)

156. Have you ever tried to quit using **marijuana**, but couldn't?

- Yes (1)
- No (0)

157. Are you planning to quit using **marijuana** in the next **6 months**?

- O Yes (1)
- O No (2)
- O Already quit (3)

158. Are you planning to quit using **marijuana** in the next **30 days**?

- O Yes (1)
- O No (0)

159. Have you ever spoken to a doctor or pharmacist about wanting to quit using **marijuana?**

- O Yes, a doctor (1)
- O Yes, a pharmacist (2)
- O Yes, both a doctor and a pharmacist (3)
- O No (0)

For the next set of questions, please read each statement and then decide how much each applied to you in the past week. In the past **7 days**, please rate how often...

	Never (1)	Rarely (2)	Sometimes (3)	Usually (4)	Always (5)
160. I felt worthless	O	O	O	O	O
161. I felt helpless	O	O	O	O	O
162. I felt depressed	O	O	O	O	O
163. I felt hopeless	O	O	O	O	O

In the **past 7 days**:

	Never (1)	Rarely (2)	Sometimes (3)	Usually (4)	Always (5)
164. I felt fearful	O	O	O	O	O
165. I found it hard to focus on anything other than my anxiety	O	O	O	O	O
166. My worries overwhelmed me	O	O	O	O	O
167. I felt uneasy	O	O	O	O	O

168. How do you describe yourself? (Mark one answer)

- O Male (1)
- O Female (2)
- O Female-to-Male (FTM)/Transgender Male/Trans Man (3)
- O Male-to-Female (MTF)/Transgender Female/Trans Woman (4)
- O Genderqueer, neither exclusively male nor female (5)
- O Additional gender category, please specify: (6) ____________________
- O Decline to answer (7)

169. What sex were you assigned at birth on your original birth certificate (check one):

- O Male (1)
- O Female (2)
- O Decline to answer (3)

170. How old are you, in years? (please select a number from the pull-down menu below)

▼ 18 (18) ... 101 or older (101)

171. Are you of Hispanic or Latino Origin?

- O No, not of Hispanic or Latino origin (0)
- O Yes, I am of Hispanic or Latino origin (1)

172. I am:

- ▢ Central American (1)
- ▢ Mexican, Mexican American, Chicano (2)
- ▢ South American (please specify) (3) ________________________________
- ▢ Another Hispanic, Latino, or Spanish origin (4)

173. I am:

- ☐ Guatemalan (1)
- ☐ Nicaraguan (2)
- ☐ Panamanian (3)
- ☐ Salvadoran (4)
- ☐ Other (5) ____

174. I am:

- ☐ Cuban (1)
- ☐ Puerto Rican (2)
- ☐ Other: (3) ____

175. Additionally, do you identify as (please check all that apply; For those who identify as Hispanic/Latino, please indicate any other categories you might identify with):

- ☐ African-American/Black (1)
- ☐ American Indian/Alaska Native (2)
- ☐ Middle Eastern (for example: Afghani, Syrian, Persian, Yemeni) (3)
- ☐ East Asian (Chinese, Korean, Japanese) (4)
- ☐ Southeast Asian (Filipino, Laotian, Vietnamese, Hmong, Mien) (5)
- ☐ South Asian (Indian, Pakistani, Sikh, Sri Lankan) (6)
- ☐ Other Asian: (7) ____
- ☐ Caucasian/White (8)
- ☐ Pacific Islander/Native Hawaiian (9)

☐ Other (10)

176. What is your current relationship status?

- O Single (never married) (1)
- O In a relationship, not living with partner (2)
- O Married/Living with partner (3)
- O Divorced/Separated (4)
- O Widowed (5)

177. What is the highest level of education you have completed?

- O Less than High School (1)
- O High School or GED (2)
- O Some college or Associates Degree (3)
- O Bachelor's Degree (4)
- O Graduate or Professional Degree (MA/JD/MD/PhD) (5)

178. What is your employment status?

- O Full-time (1)
- O Part-time (2)
- O Seeking opportunities currently (3)
- O Unemployed and not seeking opportunities (4)
- O Retired (5)

179. Were you born in the United States?

- No (0)
- Yes (1)

180. What is your preferred language?

- English (1)
- Hmong (2)
- Laotian (3)
- Spanish (4)
- Punjabi (5)
- Tagalog (6)
- Other (7) ____

181. How well do you speak English?

- Very Well (1)
- Well (2)
- Not well (3)
- Not at all (4)

182. Including yourself, how many people live in your household?

▼ 1 (1) ... 20+ (20)

183. What is your yearly **household** income (not including pensions/federal/state assistance)?

- O Up to $10,000 (1)
- O $11,000-$25,000 (2)
- O $25,001- $50,000 (3)
- O $50,001 - $75,000 (4)
- O $75,001 - $100,000 (5)
- O $100,001-$200,000 (6)
- O Over $200,000 (7)
- O I do not know (8)

184. Growing up, did your parents own or rent your home?

- O Own (1)
- O Rent (2)

185. Would you say your physical health is...

- O Excellent (5)
- O Very good (4)
- O Good (3)
- O Fair (2)
- O Poor (1)

Supplemental Material 7

STROBE Statement—Checklist of items that should be included in reports of cross-sectional studies

	Item No	Recommendation	Reported on Page #
**Title and abstract**	1	(*a*) Indicate the study's design with a commonly used term in the title or the abstract	1
		(*b*) Provide in the abstract an informative and balanced summary of what was done and what was found	2
**Introduction**			
Background/rationale	2	Explain the scientific background and rationale for the investigation being reported	3-5
Objectives	3	State specific objectives, including any prespecified hypotheses	5-6
**Methods**			
Study design	4	Present key elements of study design early in the paper	6-12
Setting	5	Describe the setting, locations, and relevant dates, including periods of recruitment, exposure, follow-up, and data collection	6
Participants	6	(*a*) Give the eligibility criteria, and the sources and methods of selection of participants	6
Variables	7	Clearly define all outcomes, exposures, predictors, potential confounders, and effect modifiers. Give diagnostic criteria, if applicable	7-10
Data sources/ measurement	8*	For each variable of interest, give sources of data and details of methods of assessment (measurement). Describe comparability of assessment methods if there is more than one group	7-10
Bias	9	Describe any efforts to address potential sources of bias	7-12
Study size	10	Explain how the study size was arrived at	6
Quantitative variables	11	Explain how quantitative variables were handled in the analyses. If applicable, describe which groupings were chosen and why	10-12
Statistical methods	12	(*a*) Describe all statistical methods, including those used to control for confounding	10-12
		(*b*) Describe any methods used to examine subgroups and interactions	10-12
		(*c*) Explain how missing data were addressed	10
		(*d*) If applicable, describe analytical methods taking account of sampling strategy	10-12
		(*e*) Describe any sensitivity analyses	10-12
**Results**			
Participants	13*	(a) Report numbers of individuals at each stage of study—eg numbers potentially eligible, examined for eligibility, confirmed eligible, included in the study, completing follow-up, and analysed	6, 10
		(b) Give reasons for non-participation at each stage	6, 10
Descriptive data	14*	(a) Give characteristics of study participants (eg demographic, clinical, social) and information on exposures and potential confounders	12, Supplemental Material 2
		(b) Indicate number of participants with missing data for each variable of interest	6,10
Outcome data	15*	Report numbers of outcome events or summary measures	12
Main results	16	(*a*) Give unadjusted estimates and, if applicable, confounder-adjusted estimates and their precision (eg, 95% confidence interval). Make clear which confounders were adjusted for and why they were included	12-14
		(*b*) Report category boundaries when continuous variables were categorized	12-14

Other analyses	17	Report other analyses done—eg analyses of subgroups and interactions, and sensitivity analyses	13-14
**Discussion**			
Key results	18	Summarise key results with reference to study objectives	14-15
Limitations	19	Discuss limitations of the study, taking into account sources of potential bias or imprecision. Discuss both direction and magnitude of any potential bias	17-18
Interpretation	20	Give a cautious overall interpretation of results considering objectives, limitations, multiplicity of analyses, results from similar studies, and other relevant evidence	18
Generalisability	21	Discuss the generalisability (external validity) of the study results	17-18
**Other information**			
Funding	22	Give the source of funding and the role of the funders for the present study and, if applicable, for the original study on which the present article is based	Title page

*Give information separately for exposed and unexposed groups.

Note: An Explanation and Elaboration article discusses each checklist item and gives methodological background and published examples of transparent reporting. The STROBE checklist is best used in conjunction with this article (freely available on the Web sites of PLoS Medicine at http://www.plosmedicine.org/, Annals of Internal Medicine at http://www.annals.org/, and Epidemiology at http://www.epidem.com/). Information on the STROBE Initiative is available at www.strobe-statement.org.
